# The ribosomal A-site finger is crucial for binding and activation of the stringent factor RelA

**DOI:** 10.1093/nar/gky023

**Published:** 2018-01-30

**Authors:** Pavel Kudrin, Ievgen Dzhygyr, Kensuke Ishiguro, Jelena Beljantseva, Elena Maksimova, Sofia Raquel Alves Oliveira, Vallo Varik, Roshani Payoe, Andrey L Konevega, Tanel Tenson, Tsutomu Suzuki, Vasili Hauryliuk

**Affiliations:** 1 University of Tartu, Institute of Technology, Nooruse 1, 50411 Tartu, Estonia; 2 Department of Molecular Biology, Umeå University, Building 6K, 6L, SE-901 87 Umeå, Sweden; 3 Laboratory for Molecular Infection Medicine Sweden (MIMS), Umeå University, Building 6K and 6L, SE-901 87 Umeå, Sweden; 4 Department of Chemistry and Biotechnology, Graduate School of Engineering, University of Tokyo, 7-3-1 Hongo, Bunkyo-ku, Tokyo 113-8656, Japan; 5 Petersburg Nuclear Physics Institute named by B.P. Konstantinov of National Research Centre “Kurchatov Institute”, Gatchina 188300, Russia; 6 Peter the Great St. Petersburg Polytechnic University, Saint Petersburg 195251, Russia; 7 National Research Centre “Kurchatov Institute”, Moscow 123182, Russia

## Abstract

During amino acid starvation the *Escherichia coli* stringent response factor RelA recognizes deacylated tRNA in the ribosomal A-site. This interaction activates RelA-mediated synthesis of alarmone nucleotides pppGpp and ppGpp, collectively referred to as (p)ppGpp. These two alarmones are synthesized by addition of a pyrophosphate moiety to the 3′ position of the abundant cellular nucleotide GTP and less abundant nucleotide GDP, respectively. Using untagged native RelA we show that allosteric activation of RelA by pppGpp increases the efficiency of GDP conversion to achieve the maximum rate of (p)ppGpp production. Using a panel of ribosomal RNA mutants, we show that the A-site finger structural element of 23S rRNA helix 38 is crucial for RelA binding to the ribosome and consequent activation, and deletion of the element severely compromises (p)ppGpp accumulation in *E. coli* upon amino acid starvation. Through binding assays and enzymology, we show that *E. coli* RelA does not form a stable complex with, and is not activated by, deacylated tRNA off the ribosome. This indicates that in the cell, RelA first binds the empty A-site and then recruits tRNA rather than first binding tRNA and then binding the ribosome.

## INTRODUCTION

Guanosine pentaphosphate (pppGpp) and tetraphosphate (ppGpp) are ubiquitous bacterial intracellular signaling nucleotides that regulate metabolism, virulence, stress and antibiotic tolerance (for review see (1–3)). The intracellular levels of pppGpp and ppGpp (collectively referred to as (p)ppGpp) are controlled by RelA/SpoT Homologue (RSH) proteins, which synthetize (p)ppGpp by transferring the pyrophosphate group of ATP onto the 3′ of GDP or GTP, and degrade (p)ppGpp by removing the 3′ pyrophosphate moiety (4).

**Figure 1.**
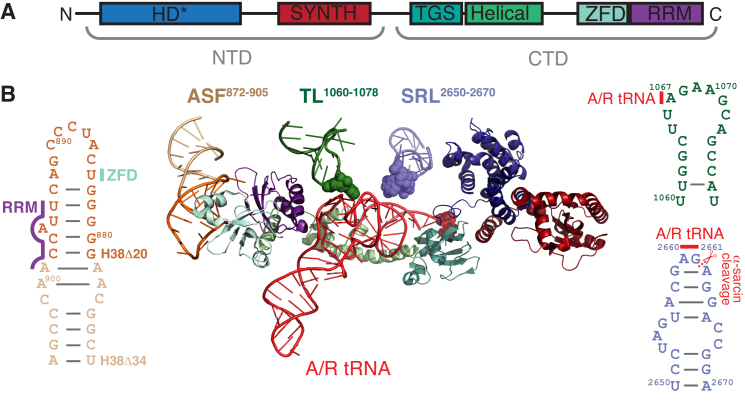
RelA’s interactions with ribosomal 23S rRNA and A/R-tRNA. RelA is a multi-domain enzyme comprised of N-terminal enzymatic half (NTD) and C-terminal regulatory half (CTD) (**A**). The NTD contains enzymatically inactive (p)ppGpp hydrolysis (HD) and a functional (p)ppGpp synthesis (SYNTH) domains, whereas the TGS (ThrRS, GTPase and SpoT), Helical, ZFD (Zinc Finger Domain) and RRM (RNA recognition motif) domains together comprise the CTD. Ribosome-bound RelA makes extensive contacts with 23S rRNA and deacylated A/R tRNA (**B**). Ribosomal A-site Finger (ASF, dark and pale orange) contacts ZFD (pale cyan) and RRM (purple) domains. Thiostrepton loop (TL, dark green) and Sarcin-Ricin Loop (SRL, slate blue) contact the A/R tRNA via rRNA residues A1067 and A2660/G2661, respectively. The TGS domain of RelA (teal) directly binds A/R tRNA’s 3′-CCA end. 23S rRNA residues A1067 and A2660/G2661, as well as A/R tRNA 3′-CCA are shown as spheres. ASF truncation H38Δ20 is highlighted in darker orange and the H38Δ34 truncation comprises the full length of the 34 nt ASF oligo shown on the figure. The 3D structure is as per from Loveland and colleagues (9) RDB accession number 5KPX, 23S rRNA secondary structure is presented as per (66).

*Escherichia coli* RelA is the most well-studied ribosome-associated RSH enzyme. The N-terminal enzymatic half of the protein consists of a catalytically active (p)ppGpp synthesis SYNTH domain and inactive (p)ppGpp hydrolysis HD domain (Figure 1A). The C-terminal regulatory half is made up of four domains: TGS (ThrRS, GTPase and SpoT), Helical, ZFD (Zinc Finger Domain; equivalent to CC, conserved cysteine as per (4)) and RRM (RNA recognition motif; equivalent to ACT, aspartokinase, chorismate mutase and TyrA, as per (4)). RelA is the subject of multifaceted allosteric regulation. Deacylated tRNA in the ribosomal A-site signals amino acid starvation and dramatically induces (p)ppGpp synthesis by RelA (5). This activation requires disengagement of the auto-inhibitory C-terminal domains (6–11). In the test tube, RelA efficiently uses both GDP and GTP as substrates, possibly with a moderate preference for GDP (12–14), while in the cell during acute stringent response the predominantly accumulated product is ppGpp (15,16). The ppGpp activates RelA at low concentrations (up to 200 μM) and inhibits at high (IC_50_ of 0.7 ± 0.4 mM) (17,18); the effects of pppGpp have not been reported. Activation of RelA by deacylated A-site tRNA is potently inhibited by the antibiotic thiostrepton and—to a much lesser extent—tetracycline (5,19–21).

Recent cryoelectron microscopy studies have provided a structural explanation for RelA’s allosteric regulation by the ribosome and deacylated A-site tRNA (Figure 1B) (9–11). The C-terminal half of the protein is buried deep inside the ribosomal complex, wrapped around the distorted A-site tRNA, the so-called A/R tRNA. The A/R tRNA makes several contacts with the ribosomal 23S rRNA: the acceptor stem contacts the sarcin-ricin loop (SRL) and the tRNA elbow region forms a stacking interaction with A1067 residue of the L7/L12 stalk rRNA base, the so-called thiostrepton loop (TL). RelA’s ZFD and RRM domains interact with the A-site finger (ASF) of 23S rRNA and the TGS domain wraps around the 3′-CCA end of A/R tRNA.

The SRL, the TL and the ASF have important roles in ribosomal functionality. SRL is a part of the GTPase-associated center—the binding site for translational factors belonging to the translational GTPase family (22). As well as being crucial for activation of GTPase activity (23,24), the SRL serves as an affinity point for their association with the ribosome (25). The isolated SRL rRNA oligonucleotide fragment binds to translational GTPases EF-G and IF2 with μM-range affinity, and complex formation is abolished by GDP (26,27). The SRL is targeted by toxins alpha-sarcin and ricin—the namesakes of this functional element—that inactivate the ribosome by cleaving the phosphodiester bond between G2661–2662 and depurinating A2660, respectively (28,29). The TL is also a part of the GTPase-associated center and is targeted by thiopeptide antibiotics thiostrepton and nosiheptide (30), with the residue A1067 being the crucial determinant for thiostrepton resistance (31,32). Finally, the ASF forms the B1a intersubunit bridge that upon ribosomal ratcheting changes its interaction partner on the small subunit from ribosomal protein S13 (non-ratcheted state) to S19 (ratcheted state) (33), and is implicated in the mechanics of EF-G-driven ribosomal translocation (34–36). Specifically, the ASF attenuates the motion of the A-site tRNA body from its classical (A/A) to hybrid (A/P) position, and its truncation promotes the formation of the so-called H1 hybrid state (P/E, A/P) (37).

Guided by recent structural insights (9–11), we have applied a combination of biochemical and microbiological techniques to probe RelA’s molecular mechanism to establish structure–functional relationships. With the exception of one study using an N-terminally tagged construct from the ASKA library (38,39), earlier biochemical studies that were performed with recombinantly produced *Escherichia coli* RelA relied on C-terminal His_6_-tagging for purification (20,21,26). Given the location of RelA’s C-terminus deep inside the ribosomal complex, it is likely that the C-terminal His_6_ tag affects the functionality of the protein causing experimental artifacts. Therefore, we have developed a procedure for purification of native, untagged *E. coli* RelA. Since it was recently shown that pppGpp is a dramatically stronger activator of the single-domain RSH RelQ than ppGpp (40–42), we characterized the interplay between RelA’s substrates (GDP and GTP) and products (ppGpp and pppGpp) in a reconstituted biochemical system. While recent cryo-EM reconstructions have generated numerous functional predictions, e.g. that the ASF is crucial for RelA’s functionality (9,10), the role of the ribosomal elements contacting RelA and A/R tRNA has not been tested in functional assays. Therefore, we took advantage of *E. coli* strain Δ7 KT101 that lacks chromosomally encoded rRNA operons (43,44) to generate *E. coli* strains with ribosomes mutated in the ASF, TL and SRL structural elements to test structure-function relationships in biochemical and microbiological assays.

## MATERIALS AND METHODS

### Construction of 23S rRNA mutants

The *E. coli* Δ7 *prrn* strain KT101 (44) harboring the pRB101 plasmid encoding the *rrnB* operon and *sacB* as a counter selectable marker (45) was used to generate a series of strains containing mutations in the 23S rRNA. Mutations in 23S rRNA were introduced in pRB103 using the QuickChange site-directed mutagenesis (Agilent Technologies) using the following sets of primers: H38Δ20: forward 5′-gggggtagagcactgtttcggcaaaacccgatgca-3′, reverse 5′-ggtattcgcagtttgcatcgggttttgccgaaaca-3′; H38Δ34: forward 5′-ctccgggggtagagcactgtttgcaaactgcgaataccggagaatg-3′, reverse 5′-cattctccggtattcgcagtttgcaaacagtgctctacccccggag-3′; A1067U: forward 5′-cagccaggatgttggctttgaagcagccatcatttaa-3′, reverse 5′-ttaaatgatggctgcttcaaagccaacatcctggctg-3′; Δ1067A: forward 5′-cagccaggatgttggcttgaagcagccatcatttaa-3′, reverse 5′-tttaaatgatggctgcttcaagccaacatcctggctg-3′; and A2660C/G2661A: forward 5′-gggctgctcctagtacgcaaggaccggagtggacgc-3′, reverse 5′-tgcgtccactccggtccttgcgtactaggagcagcc-3′. The pRB101 plasmid in KT101 was replaced by plasmid pRB103 harboring the *rrnB* corresponding mutation to yield a series of KT103 strains as per Kitahara and collegues (44). The resultant KT101-based strains were grown at 37°C with vigorous shaking in LB (Lennox) supplemented with 50 μg/ml of selective maker zeocin (phleomycin D1).

### Preparation of untagged *E. coli* RelA

All cloning was performed by the Protein Expertise Platform at Umeå University. The *relA* gene was PCR amplified from the *E. coli* DH5α strain using 5′-gtaaccATGGTTGCGGTAAGAAGTGCAC-3′ (forward) and 5′-gtaccctcaggTCAACTCCCGTGCAACCG-3′ (reverse) primers and cloned into pET24d vector using Eco81I and NcoI restriction sites, yielding plasmid RelA_pET24d. Since cutting the plasmid with Eco81I and NcoI removes the optional C-terminal His_6_ Tag sequence, RelA is expressed in native untagged form.

10 ml of LB medium supplemented with kanamycin (final concentration 50 μg/ml) was inoculated with *E. coli* BL21 DE3 transformed with RelA_pET24d and grown at 37°C during the day. The culture was used to inoculate 2× of 100 mL of ZYM 5052 medium (1:1000 dilution, 100 μl per 100 ml of media) supplemented with Trace element stock solution (1000×) (1:1000 dilution, 100 μl per 100 ml of media) and kanamycin (100 μg/ml, final concentration) (46). The culture was grown at 30°C overnight with shaking (17 h), and the cells were harvested by centrifugation in a JLA 10.500 rotor at 5000 rcf at 4°C for 12 min.

For purification, cells were resuspended in 20–40 of loading buffer (buffer A) (25 mM Tris pH 7.6, 100 mM NaCl, 2 mM MgCl_2_, 1 mM β-mercaptoethanol), pelleted at 4000 rcf for 10 min in a tabletop centrifuge, supernatant discarded, and the cell pellet taken up again in 20 ml of 20–40 of loading buffer supplemented with PMSF and DNase I added to the final concentration of 1 mM and 1 u/ml, respectively. Cells were lysed using a Stansted Fluid Power SPCH ultra high-pressure cell disrupter/homogenizer (1.2 bar), and cell debris was removed by centrifugation (35 000 rpm, 35 min at 4°C, Ti 45 rotor, Beckman). All the following steps were performed at room temperature to avoid protein precipitation. Clarified cell lysate was loaded onto a 20 ml HiPrep Q XL 16/10 (GE Healthcare) pre-equilibrated with loading buffer A. The column was washed with six CV of loading buffer, and then the protein was eluted with a gradient (3.5 CV, 0–50% buffer B) of elution buffer, buffer B (loading buffer supplemented with NaCl to final concentration of 1.5 M: 25 mM Tris pH 7.6, 1.5 M NaCl, 2 mM MgCl_2_, 1 mM β-mercaptoethanol). Fractions most enriched in RelA (≈20–25% buffer B) were pooled, totaling approximately 5 ml. The sample was loaded on HiLoad 16/600 Superdex 200 PG (GE Healthcare) equilibrated with high salt buffer, buffer C (1 M KCl, 25 mM HEPES pH 7.6, 5 mM MgCl_2_, 1 mM β-mercaptoethanol supplemented with 50 mM l-arginine and 50 mM l-glutamic acid to increase RelA’s solubility (47)). The fractions containing RelA were pooled and diluted with dilution buffer (25 mM HEPES pH 7.6, 5 mM MgCl_2_, 1 mM β-mercaptoethanol, 50 mM l-arginine, 50 mM l-glutamic acid) ∼10-fold to conductivity around 14 mSm/cm (corresponds to ∼100 mM of KCl), and loaded on 5 ml HiTrap Heparin HP column (GE Healthcare) equilibrated with 3CV of low salt buffer, buffer D (0.1 M KCl, 25 mM HEPES pH 7.6, 5 mM MgCl_2_, 1 mM β-mercaptoethanol, 50 mM L-arginine, 50 mM L-glutamic acid). Heparin sepharose acts both as an affinity and a cation exchanger (48). RelA was eluted with 6CV of 0–100% gradient of high salt buffer C. The peak containing RelA (≈25% buffer C) was collected and concentrated in Amicon Ultra (Millipore) centrifugal filter device (cut-off 10 kDa) in a storage buffer containing 0.7 M KCl, 25 mM HEPES pH 7.6, 5 mM MgCl_2_, 1 mM β-mercaptoethanol, 50 mM l-Arg, 50 mM l-Glu and 10% glycerol. The concentration of the protein was measured using Bio-Rad Protein Assay (Bio-Rad), protein aliquoted per 15 μl in PCR tubes, frozen in liquid nitrogen and stored at –80°C. The purity of the protein was confirmed by SDS-PAGE (≥90% estimated by ImageJ (49)) and the OD_280_/OD_260_ ratio of 1.8–1.9.

### Biochemical assays

RelA and EF-G enzymatic assays were performed as per (19), pre-steady state translocation kinetics was monitored as per (50), and the spin-down assays were performed as per (17). ASF oligonucleotide (5′-UCGGCAAGGGGGUCAUCCCGACUUACCAACCCGA-3′) was purchased from Metabion (HPLC purification grade). Labelling of tRNA^Phe^ with proflavine yielding Phe-tRNA^Phe^(Prf16/17) and labeling of initiator fMet-tRNA_i_^fMet^ with BODIPY-FL (Bpy) yielding Bpy-fMet-tRNA_i_^fMet^ was performed as described earlier (50,51). Pre-translocation (PRE) ribosomal complexes were enzymatically assembled on model mRNA encoding for MetPhe dipeptide (5′-GGCAAGGAGGUAAAUAAUGUUCGUUACGAC-3′) as per (52) and purified by size-exclusion chromatography on a Biosuite 450 HR 5 mm column (Waters). To estimate the IC_50_ and EC_50_ values the titration data were fitted with the 4PL model, or the Hill equation, *Y* = (*a* - *d*)/(1 + (*X/c*)*^b^*) + *d* as per (53).

Unless stated otherwise, RelA and EF-G enzymatic assays were performed at 37°C in HEPES:Polymix buffer at 5 mM Mg^2+^ (54). To assemble ‘starved’ ribosomal complexes, RNA species were sequentially added to 0.5 μM 70S in the presence or absence of alarmone nucleotides: first 2 μM mRNA(MF) 5′-GGCAAGGAGGUAAAAAUGUUCAAA-3′, followed by 2 μM P-site tRNA_i_^fMet^ and, finally, 2 μM A-site tRNA^Phe^ (Chemical Block). The reaction mixture was incubated for 2 min at 37°C after addition of each of the RNA species. While A- and P-site tRNA occupancy of the complexes was not specifically assessed to determine the fraction of ribosomal complexes lacking the A-site tRNA, these complexes are poor activators of RelA and would not significantly contribute to total pppGpp/ppGpp synthesis (18).

Once the ‘starved’ complex was assembled, 30 nM RelA supplemented with 300 μM ^3^H GDP or ^3^H GTP (Hartmann or American Radiolabeled Chemicals) was added and after an additional incubation for 2 min the reaction was started by the addition of ATP to the final concentration of 1 mM. 5 μl aliquots taken throughout the time course of the reaction and quenched with 4 μl 70% formic acid supplemented with a cold nucleotide standard (10 mM GDP and 10 mM GTP) for UV-shadowing after resolution on PEI-TLC plates (Macherey-Nagel). Nucleotides were resolved in 0.5 KH_2_PO_4_ pH 3.5 buffer, the plates dried, cut into sections as guided by UV-shadowing, and ^3^H radioactivity quantified by scintillation counting in Optisafe-3 (Fisher) scintillation cocktail. All experiments with thiostrepton and nosiheptide were performed in the presence of 0.1% (wt/vol) Pluronic F-127 that efficiently solubilizes thiopeptides (19). The GTPase activity of 0.1 μM EF-G was assayed in the presence of 300 μM ^3^H GTP and 0.5 μM 70S using TLC analysis as described above for RelA.

Pre-steady state kinetic analysis of single-round EF-G-catalyzed translocation was followed at 37°C in a buffer containing 50 mM Tris-HCl, pH 7.5, 70 mM NH_4_Cl, 30 mM KCl and 7 mM MgCl_2_. Using SX20 stopped-flow spectrometer (Applied Photophysics) 62 μl of 0.14 μM fluorescently labelled PRE 70S ribosomal complexes were rapidly mixed with an equal volume of *E. coli* EF-G at increasing concentrations (up to 10 μM), fluorescence of either proflavine or BODIPY-FL was excited at 465 nm, and the emission signal was recorded after passing the GG495 cut-off filter (Applied Photophysics). Kinetic curves were obtained by averaging 5–10 individual traces, each containing >1000 time points, and fitted to either a single-exponential (*F* = *F*_∞_ + *A*_1_*exp(–*k*_app1_*t)) or double-exponential (*F* = *F*_∞_ + *A*_1_*exp(–*k*_app1_*t) + *A*_2_*exp(–*k*_app2_*t)) model using Prism 6 (GraphPad Software Inc). Concentration dependencies were analyzed using hyperbolic fit (Michaelis–Menten).

### Microbiological assays

HPLC-based nucleotide quantification was performed as per (15). SMG plates contained M9 minimal media supplemented with 1.5% agar and 0.4% glucose as well as serine, methionine and glycine at 1 mM each (55). The plates were incubated at 37°C and scored after 48 h. To perform serial dilution spotting assays bacteria were pregrown in liquid LB (Miller) medium supplemented with zeocin for 20 h at 37°C, diluted with fresh LB to OD_600_ ranging from 10^−1^ to 10^−6^ and spotted on LB plates. The plates were scored after 12 h (35°C, 37°C, 40°C and 42°C) or 21 h (30°C and 32°C) of incubation.

## RESULTS

### Characterization of untagged native *E. coli* RelA

We characterized the enzymatic activity of untagged RelA in a biochemical system from purified *E. coli* components that we set up previously (26). Untagged *E. coli* RelA was overexpressed in *E. coli* and purified using a combination of anion exchange, gel filtration and heparin chromatography. The catalytic activity was followed in the absence of 70S ribosomes as well as the presence of either vacant 70S or ‘starved’ ribosomal complexes programmed with model mRNA(MF) encoding Met-Phe and deacylated tRNA_i_^Met^ (P-site) and tRNA^Phe^ (A-site), using either 0.3 mM ^3^H GDP or ^3^H GTP and 1 mM ATP as substrates, and upon addition of either ppGpp or pppGpp to a final concentration of 100 μM.

**Figure 2.**
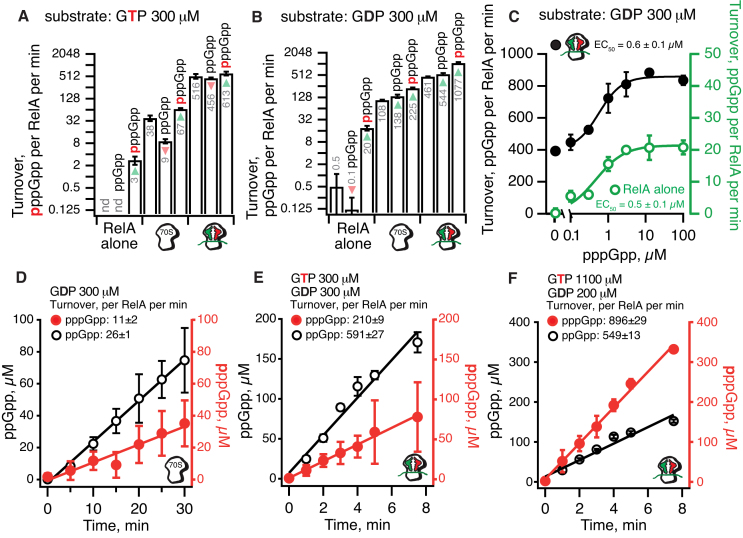
Allosteric regulator pppGpp and substrate GDP synergize for maximum enzymatic activity of *E. coli* RelA. Enzymatic activity of 30 nM RelA assayed in the presence of 1 mM (**A**-**C**) or 4 mM (**D**-**F**) ATP and 0.3 mM of either ^3^H-labelled GTP (**A**) or GDP (**B** and **C**); in the presence of equal amounts of GDP and GTP (0.3 mM each) (**D** and **E**); or in the presence of 0.2 mM GDP and 1.1 mM GTP (**F**). In the case of **D**–**F** both ^3^H-ppGpp and ^3^H-pppGpp were synthesized in the reaction. As indicated on the figure, the reaction mixtures were supplemented with combinations of 0.5 μM vacant 70S or ‘starved’ ribosomal complexes programmed with 2 μM model mRNA(MF) encoding Met-Phe and deacylated tRNA_i_^Met^ (2 μM; P-site) and tRNA^Phe^ (2 μM; A-site) as well as ppGpp or pppGpp added either 100 μM (**A** and **B**) or varying concentrations (**C**). All experiments were performed in HEPES:Polymix buffer, pH 7.5 at 37°C in the presence of 5 mM Mg^2+^. Error bars represent SDs of the turnover estimates by linear regression and each experiment was performed at least three times.

As expected, while vacant 70S ribosomes alone do have an activating effect on RelA, full activation (≈1000 times increase in turnover) is achieved only in the presence of ‘starved’ ribosomal complexes (Figure 2AB). However, unlike the C-terminal His_6_ protein that we have used previously (17,19,26), the untagged RelA is near-insensitive to the addition of 100 μM ppGpp. When GTP is used as a substrate, ppGpp acts as a weak inhibitor (Figure 2B) and when GDP is used as a substrate, the alarmone has a mild activating effect (Figure 2A). Addition of pppGpp, on the other hand, results in a pronounced effect. When GDP is used as a substrate, 100 μM pppGpp increases the turnover rate approximately twice both in the presence of 70S and ‘starved’ complexes (Figure 2B); weaker activation is observed in the presence of the GTP substrate (Figure 2A). The maximum turnover rate of 1077±62 ppGpp per RelA per minute is achieved when GDP as a substrate synergizes with pppGpp as an activator. This behavior is analogous to what we observed for the single-domain ribosome-independent RSH RelQ (41). To quantitatively characterize RelA’s sensitivity to pppGpp, we titrated the alarmone in the presence of ^3^H GDP substrate. Already at 2 μM, pppGpp exerts its full activating effect, both on RelA alone or RelA in the presence of ‘starved’ ribosomal complexes (Figure 2C).

In the cell both of RelA’s substrates—GTP and GDP—are always present. To mimic cellular conditions, we tested RelA’s synthetic activity upon simultaneous addition of ^3^H GDP and ^3^H GTP. We used equal amounts of both substrates (0.3 mM), omitting both ppGpp or pppGpp since both alarmones will be synthesized *in situ* and already 1% conversion of ^3^H GTP to ^3^H pppGpp will generate enough alarmone to fully activate RelA. Activated by either vacant 70S (Figure 2D) or ‘starved’ ribosomal complexes (Figure 2E) RelA uses GDP approximately two times more efficiently, in good agreement with the experiments with individual nucleotide substrates (Figure 2AB). However, in exponentially growing *E. coli*, GTP is present in five times excess over GDP, i.e. 1.1 mM versus 220 μM, respectively (15). Although under these conditions synthesis of pppGpp dominates the reaction (Figure 2F), the five times less abundant GDP substrate is still converted *relatively* more efficiently, as evidenced by merely twice lower turnover rate (549 ± 13_ppGpp_ versus 896 ± 29_pppGpp_ reactions per RelA per minute).

**Figure 3.**
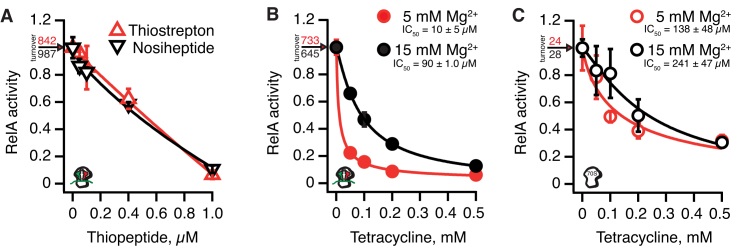
Inhibition of RelA’s enzymatic activity by antibiotics thiostrepton, nosiheptide and tetracycline. Inhibition of RelA activated ‘starved’ ribosomal complexes by thiostrepton (red trace) or nosiheptide (black trace) in the presence of 0.1% (wt/vol) Pluronic F-127 (**A**). Inhibition of RelA activated by ‘starved’ ribosomal complexes (**B**) or vacant 70S ribosomes (**C**) by tetracycline in the presence of 5 mM (red traces) or 15 mM (black traces) Mg^2+^. Reaction mixtures contained 30 nM RelA, 100 μM ppGpp, 0.3 mM ^3^H-labelled GDP and 1 mM ATP as well as 0.5 μM of either vacant 70S or ‘starved’ ribosomal complexes programmed with 2 μM model mRNA(MF) encoding Met-Phe and deacylated tRNA_i_^Met^ (2 μM; P-site) and tRNA^Phe^ (2 μM; A-site). All experiments were performed in HEPES:Polymix buffer pH 7.5 at 37°C in the presence of 5 mM Mg^2+^ unless stated otherwise (**B** and **C**, black traces, 15 mM Mg^2+^). Uninhibited turnover values corresponding to 1.0 activity are provided on individual panels. Error bars represent SDs of the turnover estimates by linear regression and each experiment was performed at least three times.

Finally, we used our native, untagged RelA to reassess the effects of antibiotics known to inhibit RelA. Our previous experiments of the effect of antibiotics on RelA (19) were performed with a C-terminally His_6_-tagged which is, evidently, not completely equivalent with the native protein. The thiopeptides thiostrepton and nosiheptide have nM-range affinity to their ribosomal target (56). Both compounds efficiently inhibit RelA activated by ‘starved’ complexes, and the linearity of the titration curve reflects efficient sequestration of ribosomes in an inactive complex upon addition of the thiopeptide (Figure 3A). The A-site tRNA competitor tetracycline was reported to be a poor inhibitor of RelA with an IC_50_ of 0.2–0.5 mM (19,20). Surprisingly, the inhibition is almost insensitive to the presence of the A-site tRNA (19). However, earlier experiments were performed in the presence of Mg^2+^ in 15 mM concentration, which leads to non-physiologically tight binding of the A-site tRNA to the ribosome (52). Therefore, we repeated the tetracycline titrations in the presence of either ‘starved’ complexes (Figure 3B) or vacant 70S (Figure 3C) in the presence of either 5 or 15 mM Mg^2+^ (red and black traces, respectively). While in the presence of 15 mM Mg^2+^ tetracycline is, indeed, a poor inhibitor of untagged RelA, in the presence of 5 mM Mg^2+^ inhibition is significantly more efficient and is dependent on the presence of the A-site tRNA. This indicates that poor inhibition reported earlier (19,20) is likely due to non-physiologically high Mg^2+^ concentrations in the biochemical system. However, direct inhibition of RelA by antibiotics is still likely to be physiologically irrelevant. Even at 5 mM Mg^2+^ it takes 50 μM tetracycline to inhibit RelA by 80% in the test tube, while in live cells near-complete inhibition (p)ppGpp accumulation is achieved already at 3 μM tetracycline via an indirect mechanism, i.e. inhibition of translation causing aminoacylation of deacylated tRNA (19).

### Mutational analysis of ASF, SRL and TL roles in EF-G and RelA functionality in a reconstituted biochemical system

We have constructed a set of strains expressing 23S rRNA mutants from a pRB103 plasmid in the genetic background of Δ7 *E. coli* strain KT101 that lacks all seven chromosomally encoded *rrn* operons (43) and purified a panel of mutant ribosomes: (a) H38Δ34 (34) and H38Δ20 deletions in the ASF of domain II, helix H38 (b) A1067U point mutation and ΔA1067 deletion in TL of domain II, helix H43, and, finally, (c) wild type ribosomes cleaved between G2661 and A2662 with alpha-sarcin and A2660C/G2661A mutant in the SRL of domain VI, helix H95 (Figure 1B). The ribosomes were tested for activation of EF-G GTPase activity and ppGpp synthesis by RelA.

**Figure 4.**
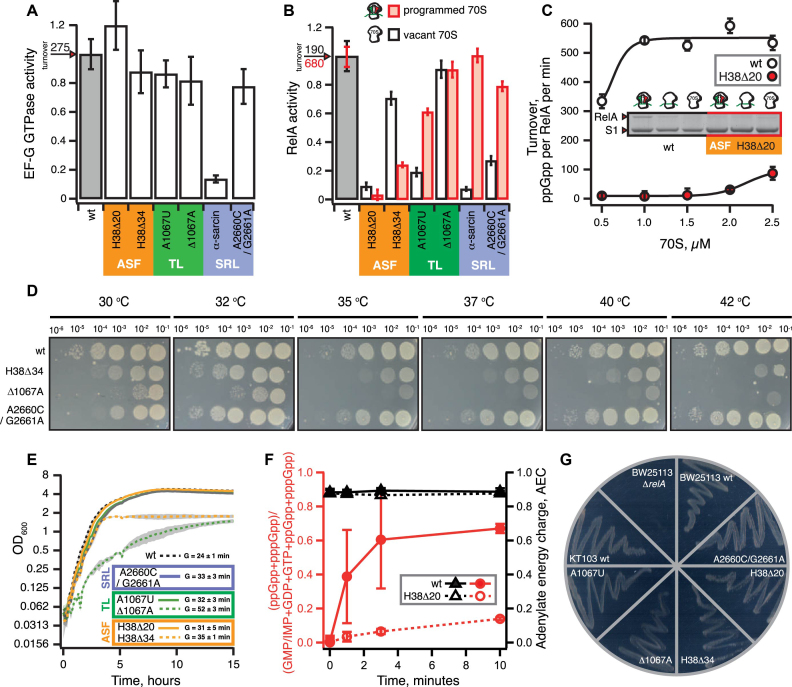
Effects of molecular perturbations of ASF, SRL and TL on RelA’s enzymatic activity. GTPase activity of 0.1 μM EF-G assayed in the presence of 0.3 mM of ^3^H GTP and 0.5 μM 70S (**A**). ^3^H-ppGpp synthesis by 30 nM RelA assayed in the presence of either 1 mM ATP and 0.3 mM ^3^H GDP as well as either 70S ribosomes (0.5 μM; black bars) or ‘starved’ ribosomal complexes (0.5 μM 70S, 2 μM mRNA(MF), tRNA_i_^Met^ and tRNA^Phe^, 2 μM each; red bars) (**B**). ^3^H-ppGpp synthesis by RelA in the presence of increasing concentrations of either wild type (empty circles) or H38Δ20 (red filled circles) 70S programmed with 2 μM mRNA(MF) as well as tRNA_i_^Met^ and tRNA^Phe^, 2 μM each (**C**). Association 1 μM RelA with either 1 μM wild type or H38Δ20 70S ribosomes programmed with 2 μM mRNA(MF) as well as tRNA_i_^Met^ and tRNA^Phe^, 2 μM each (**C**, insert). Growth of Δ7 *E. coli* KT101-based strains expressing either wild type or mutant 23S rRNA from pRB103 plasmid on LB plates at varied temperature (**D**), in the liquid LB culture at 37°C (**E**) and on SMG plates at 37°C (**G**). Serial dilution spotting analysis on LB plates was scored at 12 (35°C, 37°C, 40°C and 42°C) or 21 (30°C and 32°C) hours of incubation (**D**). Nucleotide pools in Δ7 *E. coli* KT101-based strains expressing either wild type (filled symbols) or H38Δ20 mutant (empty symbols) 23S rRNA from pRB103 plasmid grown on MOPS liquid media supplemented with 0.4% glucose and challenged by mupirocin added to final concentration of 150 μg/ml (**F**). The error bars represent SD of the mean of biological replicates (n = 3). All biochemical experiments were performed at 37°C in HEPES:Polymix buffer, pH 7.5 at 5 mM Mg^2+^. Uninhibited turnover values corresponding to 1.0 activity in the presence of wild type ribosomes are provided on individual panels A and B. Error bars represent SDs of the turnover estimates by linear regression if not stated otherwise. Each experiment was performed at least three times.

In the case of EF-G GTPase activity, the only significant effect is inhibition (≈six times) by the alpha-sarcin cleavage (Figure 4A). The ASF was earlier shown to play a role in ribosome dynamics, with deletions causing a moderate increase in translocation and puromycin reactivity (34,35,37). However, while translocation is a rapid process taking under 100 ms under single-turnover conditions (50) previously published translocation assays using ASF-truncated ribosomes either lacked necessary temporal resolution (34,35) or reported non-physiologically slow kinetics with *V*_max_ of 1.8–3.0 s^−1^ at saturating EF-G concentrations (37). Therefore, we revisited the pre-steady state kinetic analysis of single-round EF-G-catalyzed translocation on ASF-truncated (H38Δ20 and H38Δ34) 70S ribosomes using stopped-flow fluorescent assays (50). To monitor the movement of A-site fMetPhe-tRNA^Phe^ on the 50S subunit we used either the BODIPY-FL (Bpy) label attached to the N-terminal methionine (Bpy-MetPhe-tRNA^Phe^) or the proflavine label attached to the D-loop [fMetPhe-tRNA^Phe^(Prf16/17)] (50). The combination of the two labels allows us to follow the movement of both the peptide-bound acceptor end and elbow region of the A-site peptidyl-tRNA, and kinetic uncoupling of the two signals can detect the perturbation of concerted translocation caused, for instance, by mutations in EF-G and antibiotics such as Hygromycin B, Spectinomycin or Viomycin (50). While both H38Δ20 and H38Δ34 ASF truncations do have a moderate effect (up to 15% deviation from the wild type values) on the rate of translocation at saturating EF-G concentrations as measured by the two fluorescent reporters, the translocation remains coordinated, indicating an absence of significant defects (Supplementary Figure S1).

In the case of RelA, both the A2660C/G2661A SRL mutation and alpha-sarcin cleavage inhibit activation by the 70S alone (Figure 4B). However, activation by ‘starved’ complexes is retained, suggesting that intact wild type SRL is not essential for RelA activation by A/R tRNA. Neither deletion of A1067 nor the A1067U mutation have a dramatic effect on RelA activation by ‘starved’ complexes either, but, similarly to SRL perturbations, reduce the activation by vacant 70S. Conversely, truncations of the ASF have a strong inhibitory effect on RelA. Surprisingly, the H38Δ20 deletion has a more prominent effect than the more extensive H38Δ34 truncation: activation by ‘starved’ complexes is reduced 30- and 4-fold, respectively.

According to the available structural data, the ASF is the key affinity point for RelA on the ribosome. Therefore, it is plausible that ASF truncation inhibits not the RelA activation *per se*, but only the binding to the ribosome and, thus, increasing the concentration of ribosomal complexes should restore the activation. Therefore, we titrated ribosomes in our biochemical system (Figure 4C). In the case of wild type ‘starved’ complexes, the maximum activity is achieved at 1 μM 70S. In the case of H38Δ20 ribosomes, although RelA activity does increase with increasing ribosomal concentration, it constitutes merely 20% of that of a wild type at 2.5 μM 70S. Finally, to directly demonstrate the role of ASF in RelA recruitment to the ribosome, we probed the RelA interaction with ribosomes in a spin-down assay and, indeed, RelA binds considerably poorer to the ASF-truncated ribosomes as compared to the wild type ones (Figure 4C, insert).

### Effects of ASF truncations on the stringent response functionality in live *E. coli* cells

To put the effects of the ASF truncations into a cellular context, we characterized the *E. coli* strains used to generate the mutant ribosomes. In comparison with the wild type, two strains displayed a prominent growth defect in LB medium (Figure 4DE): ΔA1067 has a significantly longer doubling time (52±3 minutes vs. 24±1 minutes) and the H38Δ34 ASF truncation mutant had an abnormal growth arrest upon reaching the OD_600_ of 1.8. Importantly, while the H38Δ20 ASF truncation near completely abolishes RelA activation in the test tube (Figure 4B), the mutant strain displays only a minor growth defect (doubling time of 31 ± 5 minutes versus 24±1 minutes of a wild type) and, just like the wild type, the stationary phase reaches OD_600_ of above 4.0. For H38Δ34, Δ1067A and A2660C/G2661A strains we complemented the liquid media grow experiments with serial dilution spotting assays performed in a range of temperatures (from 30°C to 42°C) allowing detection of cold and heat sensitivity phenotypes in addition to general growth defects (Figure 4D). While the H38Δ34 strain has a pronounced growth defect at all the temperatures tested, the effect becomes more pronounced as the temperature increases from 32°C to 42°C; the Δ1067A TL mutant behaves similarly. Finally, the A2660C/G2661A mutant grows normally at 32–42°C, is cold-sensitive and displays a minor growth defect at 30°C.

We followed the dynamics of acute stringent response in wild type and H38Δ20 strains upon addition of mupirocin (pseudomonic acid), a competitive inhibitor of isoleucine aminoacyl-tRNA synthetase to a final concentration of 150 μg/ml (57) (Figure 4F). Using an HPLC-based approach (15), we quantified the guanosine (pppGpp, ppGpp, GTP, GDP and GMP/IMP) and adenosine (ATP, ADP and AMP) nucleotide pools. The latter was used to calculate the adenylate energy charge, AEC, defined as ([ATP] + 0.5[ADP])/([ATP] + [ADP]+[AMP])—a universal quantitative measure of cellular energy levels that remains ≥0.8 in viable cells (58). In both strains the AEC remains unchanged during the course of the mupirocin treatment and is above 0.8. At the same time, the (p)ppGpp levels undergo a dramatic change. In the wild type (p)ppGpp level increases rapidly and within three minutes reaches a plateau at >60% of the total guanosine pool. The situation is different in the H38Δ20 mutant. Over the course of a 10-minute-long mupirocin treatment the (p)ppGpp fraction linearly increases, reaching about 14%. This clearly demonstrates that while RelA’s functionality is, indeed, compromised, it is not abolished and the cells can mount the stringent response. Finally, we have plated all of the mutant strains on the SMG plates – a classical microbiological test for complete loss of RelA functionality, the so-called ‘relaxed’ phenotype (55) (Figure 4G). All of the strains including the H38Δ20 mutant pass the test, again demonstrating that RelA functionality is not completely lost.

### Interaction of RelA with deacylated tRNA off the ribosome

**Figure 5.**
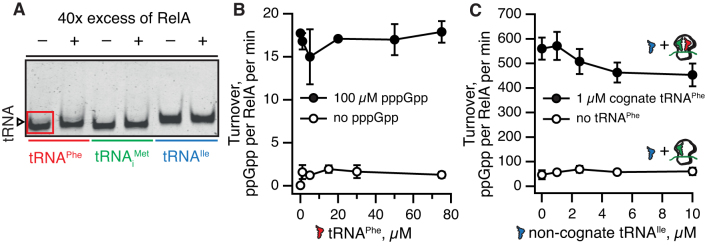
RelA does not form a stable complex with deacylated tRNA off the ribosome. No RelA:tRNA complex with either *E. coli* tRNA^Phe^, tRNA_i_^Met^ or tRNA^Ile^ is detected by EMSA, as judged by the absence of an extra band on the gel in the presence of RelA (**A**). Deacylated tRNA^Phe^ does not activate the enzymatic activity of 250 nM RelA either in the absence (empty black circles) or presence (filled black circles) of 100 μM pppGpp (**B**). Non-cognate deacylated tRNA^Ile^ neither significantly inhibits ppGpp synthesis by 50 nM RelA in the presence of ‘starved’ ribosomal complexes (filled black circles) nor activates RelA in the presence of ribosomal complexes with a vacant A-site (empty black circles) (**C**). EMSA assays were performed in the presence of 70 nM tRNA and 2.8 μM RelA (A). ^3^H-ppGpp synthesis by RelA was assayed in the presence of 1 mM ATP and 0.3 mM ^3^H GDP as well as increasing concentrations of deacylated tRNA as indicated on the figure (**B** and **C**). Ribosomal complexes were formed *in situ* by combining 0.5 μM 70S, 1 μM mRNA(MF) and 1 μM tRNA_i_^Met^ either in the presence or absence of 1 μM cognate A-site tRNA^Phe^ (**C**). Experiments with ribosomal complexes were performed in the presence of 100 μM ppGpp. All experiments were performed at 37°C in HEPES:Polymix buffer, 5 mM Mg^2+^, pH 7.5. Error bars represent SDs of the turnover estimates by linear regression and each experiment was performed at least three times.

It is plausible that multiple contacts between *E. coli* RelA and deacylated tRNA observed on the ribosome could also drive complex formation off the ribosome. Provided the structure of the binary RelA:tRNA complex is similar to that on the ribosome (Figure 1B), disengagement of RelA’s C-terminal auto-inhibitory domains would cause (partial) activation of (p)ppGpp synthesis activity. One could even envision RelA promoting the delivery of tRNA to the ribosome, acting in an EF-Tu-like manner but specific for deacylated tRNA. To test this, we used an electrophoretic mobility shift assay (EMSA) to probe tRNA interaction of *E. coli* RelA. By analogy with the rRNA SRL oligonucleotide that retains the ability to bind translational GTPases EF-G and IF2 (26,27), we also tested a 34-nucleotide long RNA corresponding to the ASF element (nucleotides 872–905 of helix H38, Figure 1B). While we failed to detect RelA binding to either tRNA (Figure 5A) or the ASF RNA (Supplementary Figure S2A) oligonucleotide, it is possible RelA does form a low-affinity complex that remains undetected by EMSA, since the method is poorly suited for studying weak interactions with *K*_D_ above 3 μM (59).

To probe these potential transient interactions, we used modulation of RelA’s enzymatic activity as a readout of the RelA:RNA complex formation. In the case of ASF, sequestration of RelA in a complex could compete with ‘starved’ ribosomal complexes and inhibit RelA. However, when added in 20× excess over ribosomes (0.5 μM versus 10 μM) the ASF oligonucleotide does not inhibit RelA’s activity (Supplementary Figure S2B). In the case of deacylated tRNA, complex formation is expected to activate RelA in the absence of ribosomes by countering auto-inhibition. However, this is not the case: the ppGpp synthesis activity of RelA, both in the absence and presence of 100 μM pppGpp, remains unchanged upon addition of up to 75 μM deacylated tRNA^Phe^ (Figure 5B). Since activation of RelA by ‘starved’ complexes is strictly dependent on the A-site tRNA being cognate to the A-site codon (5), we tested whether non-cognate tRNA^Ile^ could outcompete ‘starved’ complexes programmed with mRNA(MF) and consequently inhibit the system. However, even when added to a final concentration of 10 μM, tRNA^Ile^ does not significantly inhibit RelA (Figure 5C, filled circles). One could imagine that the absence of inhibition is due to tRNA^Ile^ binding to the vacant A-site and thus acting as an activator instead of tRNA^Phe^. This, however, is not the case, since no activation by tRNA^Ile^ is observed when we use ribosomal complexes with a vacant A-site (Figure 5C, empty circles).

Taken together, our results suggest that RelA’s recruitment to—and activation by—‘starved’ ribosomes relies on an additive combination of weak interactions with tRNA and rRNA rather than via formation of a stable binary RelA:tRNA complex off the ribosome followed by its accommodation in the vacant A-site.

## DISCUSSION

Upon acute amino acid starvation in *E. coli*, ppGpp and pppGpp levels rapidly increase and reach the maximum levels in approximately five minutes (15,16). While ppGpp is the dominant species of the two, the reason for this is rapid conversion of pppGpp to ppGpp by guanosine pentaphosphate phosphohydrolase, GPPA (60). Under balanced growth condition pppGpp levels are exceedingly low, undetectable by HPLC-based approaches (15), indicating either near-absent synthesis or efficient conversion by GPPA. While the two alarmone nucleotides are often bundled together as (p)ppGpp, they are not identical in terms of their regulatory effects. For example in *E. coli*, pppGpp is less potent than ppGpp in transcriptional regulation (61) and interacts differently with RNAP (62).

Our biochemical experiments provide insight into the enzymatic logic of RelA-mediated (p)ppGpp accumulation. A general feature of metabolic enzymes is that the affinity for substrates is in tune with substrate concentration (63). RelA has an approximately two-fold preference for the less abundant substrate, GDP (Figure 2DE). However, in the presence of cellular concentrations of substrates, pppGpp is the dominant product (Figure 2F). This is in good agreement with nucleotide measurements showing that the *gppA-*deficient strain predominantly accumulates pppGpp (60). RelA’s synthetic activity is fine-tuned by pppGpp, which stimulates production of ppGpp from GDP (Figure 2). This results in efficient conversion of the low-abundance substrate GDP to ppGpp as well as the high-abundance substrate GTP to pppGpp, thereby maximizing total alarmone production by RelA. Similar behavior has been observed for the single-domain ribosome-independent RSH enzyme RelQ (40,41). The high sensitivity of RelA to pppGpp (Figure 2C, EC_50_ of 0.5–0.6 μM) is indicative of relatively tight binding of the alarmone to RelA. This suggests structural and biochemical methods may be successful in locating the allosteric binding site.

In our earlier report using C-terminally His_6_-tagged RelA (18) we observed much stronger activation in the presence of ppGpp than what we observe now with the untagged protein. A possible explanation is that additional amino acids at the C-terminus destabilize the auto-inhibited conformation and render the protein over-sensitive to activation by the nucleotide alarmone. Note that single particle tracking investigations performed so far used a C-terminally tagged RelA since it is easier to integrate the fluorescent protein label after the *relA* ORF (64,65). This kind of tagging likely causes larger perturbations than a His_6_ tag, e.g. by clashing directly with the A/R tRNA and thus causing dissociation of RelA of the ribosome upon induction of the stringent response. Using a rigorously tested N-terminally tagged version of RelA in tracking experiments could resolve the controversy about the mechanism of RelA.

Our functional analysis has demonstrated that interaction of the 23S rRNA ASF with RelA’s ZFD domain as observed by cryo-EM (9,10) is the key for RelA binding to the ribosome and its consequent activation (Figure 4). However, even upon complete deletion of the ASF, RelA retains residual functionality both in the test tube and in live cells. Importantly, this residual activity is sufficient for the mutant strain lacking the ASF to pass the SMG test for a stringent phenotype. Therefore, the relatively crude SMG test alone is not sufficient for validation of the functionality of RelA constructs tagged with fluorescent proteins.

Finally, we show that *E. coli* RelA does not form a stable complex and is not activated by deacylated tRNA off the ribosome (Figure 5AB). The deacylated tRNA is a significantly poorer binder to RelA in comparison with ‘starved’ complexes, and non-cognate tRNA does not outcompete and inhibit activation by the latter (Figure 5C). Therefore, we favor the model of RelA mechanism proposed by Loveland and colleagues (9), where RelA first binds the empty A-site and then recruits the tRNA rather than first binding the tRNA off the ribosome. The latter interaction, while clearly possible, is too transient to serve as a key on-path intermediate for RelA’s functional cycle.

## Supplementary Material

Supplementary Data
