# Supplementary material for: The ribosomal A-site finger is crucial for binding and activation of the stringent factor RelA
Source: Nucleic Acids Res. 2018 Jan 30;46(4):1973–83. doi: 10.1093/nar/gky023 (PMC5829649; doi:10.1093/nar/gky023)
Supplement: Supplementary Data [file gky023_supp.docx]

**SUPPLEMENTARY ONLINE MATERIALS**

for

**The ribosomal A-site finger is crucial for binding and activation of the stringent factor RelA**

Pavel Kudrin^1,†^, Ievgen Dzhygyr^2,3,†^, Kensuke Ishiguro^4^, Jelena Beljantseva^1^, Elena Maksimova^5,6^, Sofia Raquel Alves Oliveira^1^, Vallo Varik^1,§^, Roshani Payoe^1^, Andrey L. Konevega^5,6,7^, Tanel Tenson^1^, Tsutomu Suzuki^4^, Vasili Hauryliuk*^,1,2,3^

^1^University of Tartu, Institute of Technology, Nooruse 1, 50411 Tartu, Estonia

^2^Department of Molecular Biology, Umeå University, Building 6K, 6L, SE-901 87 Umeå, Sweden

^3^Laboratory for Molecular Infection Medicine Sweden (MIMS), Umeå University, Building 6K and 6L, SE-901 87 Umeå, Sweden

^4^Department of Chemistry and Biotechnology, Graduate School of Engineering, University of Tokyo, 7-3-1 Hongo, Bunkyo-ku, Tokyo 113-8656

^5^Petersburg Nuclear Physics Institute named by B.P. Konstantinov of National Research Centre “Kurchatov Institute”, Gatchina 188300, Russia

^6^Peter the Great St. Petersburg Polytechnic University, Saint Petersburg 195251, Russia

^7^National Research Centre “Kurchatov Institute”, Moscow 123182, Russia

*To whom correspondence should be addressed. Tel: +46(0)706090493; Fax: +46(0)90772630; Email: [vasili.hauryliuk@umu.se](mailto:vasili.hauryliuk@umu.se)

^§^Present Address: Cellular and Molecular Pharmacology, Louvain Drug Research Institute, Université Catholique de Louvain, Brussels, Belgium

^†^These authors contributed equally to the paper as first authors

**Supplementary Figure 1 | Pre-steady state kinetics of single-round translocation of ASF-truncated PRE complexes.** To follow ribosomal translocation, pre-translocation ribosomal complexes (PRE) bearing fluorescently labeled peptidyl-tRNA with fluorophore proflavine in the D-loop of tRNA (**A**) or BODIPY (Bpy) on peptide moiety (**D**) were rapidly mixed with elongation factor EF-G and GTP in a stopped flow apparatus, and fluorescence signal was monitored over time. Time courses of proflavine (**B**) and Bpy (**E**) fluorescence change upon translocation of wild type (blue), H38Δ34 (orange) or H38Δ20 (green) PRE ribosomal complexes in the presence of a saturating concentration of EF-G (5 μM). Concentration dependencies of the rate of translocation with increasing concentrations of EF-G as monitored by the proflavine label on D-loop of tRNA (**C**; translocation rates at saturating concentrations of EF-G are 48±1 s^-1^ for H38Δ20, 35±2 s^-1^ for H38Δ34 and 42±1 s^-1^ for wild type) or BODIPY label on peptide moiety (**F**; translocation rates at saturating concentrations of EF-G are 27±3 s^-1^ for H38Δ20, 27±2 s^-1^ for H38Δ34 and 25.7±0.6 s^-1^ for wild type).

**Supplementary Figure 2 | RelA activated by ‘starved’ ribosomal complexes is refractive to inhibition by isolated ASF rRNA oligonucleotide.** The 34 nt long ASF RNA oligonucleotide (**Figure 1B**) does not inhibit RelA’s enzymatic activity (**A**) and no RelA:ASF complex is detected by EMSA even in the presence of 60x excess of the protein (**B**). EMSA assays were performed in the presence of 70 nM RNA and increasing concentrations of RelA as indicated on the figure. ^3^H-ppGpp synthesis by 30 nM RelA assayed in the presence of 1 mM ATP and 0.3 mM ^3^H-GDP, ‘starved’ ribosomal complexes (0.5 μM 70S, 2 μM mRNA(MF), tRNA_i_^Met^ and tRNA^Phe^, 2 μM each) in the presence and absence of 10 μM 34 nt ASF oligo. All experiments were performed at 37°C in HEPES:Polymix buffer, 5 mM Mg^2+^, pH 7.5. Error bars represent SDs of the turnover estimates by linear regression.
